# Correlation of CD146 expression and clinicopathological characteristics in esophageal squamous cell carcinoma

**DOI:** 10.3892/ol.2014.2227

**Published:** 2014-06-05

**Authors:** YAN LI, JIN-MING YU, XUE-MEI ZHAN, LI-LI LIU, NING JIN, YAN-XIA ZHANG

**Affiliations:** 1 Department of Radiation Oncology, Shandong Cancer Hospital, Shandong University, Jinan, Shandong 250117, P.R. China; 2 Department of Radiation Oncology, Linyi People’s Hospital, Linyi, Shandong 276000, P.R. China; 3 Department of Pathology, Linyi People’s Hospital, Linyi, Shandong 276000, P.R. China

**Keywords:** CD146, esophageal squamous cell cancer, clinicopathological characteristics, immunohistochemistry

## Abstract

CD146, a cell adhesion molecule, is found in normal and tumor tissues. The level of its expression has been found to directly correlate with tumor progression and metastatic potential. The objective of this study was to investigate the expression of CD146 in esophageal squamous cell carcinoma (ESCC) and its correlation with clinicopathological parameters. Tumor specimens were collected from 63 patients with ESCC who underwent complete resection. We analyzed the CD146 expression levels in ESCC by immunohistochemistry. The expression of CD146 was detected and it was observed to correlate with clinicopathological parameters. Sixty-three cases of normal squamous mucosa were included for comparison. CD146 expression was identified in 46.0% (29/63) of the ESCC samples, and no positive (weak to moderate or moderate to strong) expression was found in the normal squamous epithelium samples (χ^2^=27.248; P<0.0001). CD146 expression was associated with lymph node metastasis (χ^2^=5.117; P=0.024) and advanced clinical stage (χ^2^=4.661; P=0.031). CD146 expression was one of the significant predictors of survival (hazard ratio, 2.838; 95% confidence interval 1.102–7.305). The overexpression of the CD146 gene was one of the important phenotypes and characteristics in ESCC carcinomatous change. We found that CD146 expression was associated with lymph node metastasis and advanced clinical stage, and was an indicator of poor prognosis in ESCC patients. CD146 may prove to be an important tumor marker for the individualized treatment for ESCC.

## Introduction

Esophageal carcinoma is one of the most fatal types of cancer with highly aggressive potency. Due to its poor prognosis and high incidence rate, which exceeds 100 cases per 100,000 individuals per year in China (1). it is important to investigate the initiation and progression of esophageal squamous cell carcinoma (ESCC) and to study the associated prognostic factors. Numerous genes and proteins with abnormal expression and function have been investigated in the pathogenesis of ESCC, such as epidermal growth factor receptor, survivin and cyclooxygenase-2 (2–4). However, the molecular mechanisms involved in the pathogenesis of ESCC are not fully understood. CD146, a member of the immunoglobulin gene superfamily, was first identified as a cell adhesion molecule and a marker of melanoma progression and metastasis (5). It has been demonstrated that CD146 is expressed on vascular endothelium, smooth muscle and other cells in normal tissue, and mediates cation-independent adhesion through interactions with an unidentified ligand on the surface of various cells (6).

Overexpression of CD146 has been identified in a number of types of cancer, including melanoma, prostate cancer, epithelial ovarian cancer and breast cancer (7–10). Its expression levels have been found to correlate with tumor progression and metastatic potential, thus establishing CD146 as an important candidate molecule involved in tumor growth and metastasis. However, at present, studies that report CD146 expression in ESCC patients are rare. Therefore, we evaluated the expression of CD146 in ESCC and its association with clinicopathological parameters, such as clinical stage of the disease, in the present study.

## Materials and methods

### Patients and specimens

The expression of CD146 in 63 surgically resected ESCC specimens and in 63 normal esophageal mucosa samples obtained from ESCC patients was analyzed by immunohistochemistry. In total, 63 patients with ESCC underwent surgery at Linyi People’s Hospital (Linyi, China), from August 2010 to February 2012. All patients underwent total esophagectomy and radical lymph node dissection. Normal esophageal mucosal samples were taken from a region >5 cm distant from the cancer, as non-tumor control samples. There were 63 cases in the control group. Histopathological specimens were fixed in 10% buffered formalin, processed routinely and embedded in paraffin. All specimens were obtained from patients who had not received chemotherapy and radiotherapy prior to surgery. Following hematoxylin and eosin staining, all sections were reviewed and reexamined. The grade of tumor differentiation was determined according to the classification of the World Health Organization 2011 (11), and the clinical stage was according to the tumor-node-metastasis classification system of the International Union Against Cancer 2002 (12). The invasion depth was determined according to the criteria of the International Union Against Cancer 2002 (12). The study was approved by the ethics committee of Shandong Cancer Hospital of Shandong University (Shandong, China). Patients provided written informed consent.

### Immunohistochemical staining

The specimens of ESCC and non-cancerous esophageal mucosa were cut into 4- to 5-μm-thick sections and mounted onto slides, deparaffinized with xylene, and rehydrated with graded concentrations of ethanol. Endogenous peroxidase activity was blocked with 3% hydrogen peroxide (H_2_O_2_) in deionized water for 10 min. The slides were then washed three times with phosphate-buffered saline (PBS, pH 7.2–7.4) buffer for 2 min. An antigen retrieval technique was used before application of the primary antibody (10 mmol/l sodium citrate solution, pH 6.0 in a pressure cooker for 2–2.5 min). After three washes with PBS, an aliquot of 50 μl of primary antibody (rabbit anti-human CD146 monoclonal antibody; ncl-cd146; Leica Biosystems, Newcastle Upon Tyne, United Kingdom) was applied to each section and incubated at 37°C for 60 min. This was followed by washing three times with PBS, and the antibodies were detected using the secondary antibody detection kit Polink-1 PV-6000 (Zhongshan Goldenbridge Biotechnology Co., Ltd., Beijing, China). Sections were stained with 3,3′-diaminobenzidine (DAB) followed by distilled water. The sections were lightly counterstained in Haris hematoxylin solution (Zhongshan Goldenbridge Biotechnology Co., Ltd.) for microscopic examination (BX53, Olympus Corporation, Tokyo, Japan). The section were dehydration in an alcohol gradient, cleared with xylene and mounted using neutral gum.

**Figure 1:**
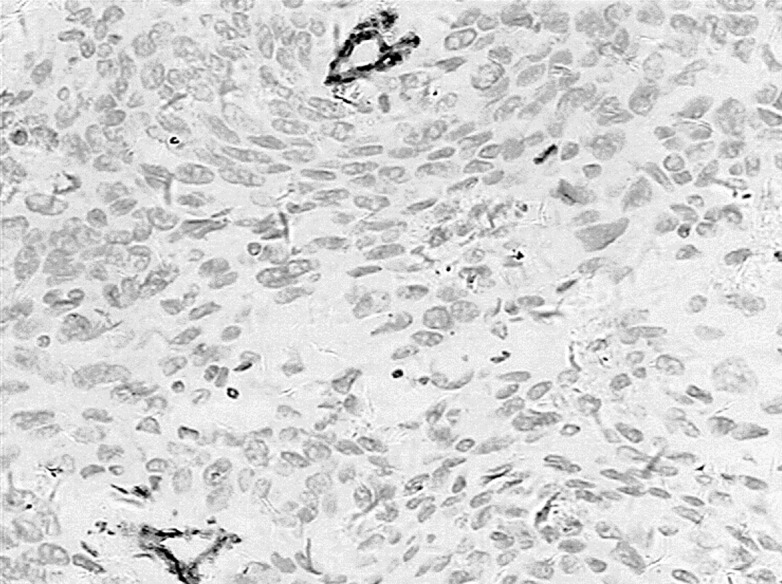
Representative image of negative immunohistochemical staining for CD146 (score of 1) in an esophageal squamous cell carcinoma specimen (magnification, ×200).

**Figure 2:**
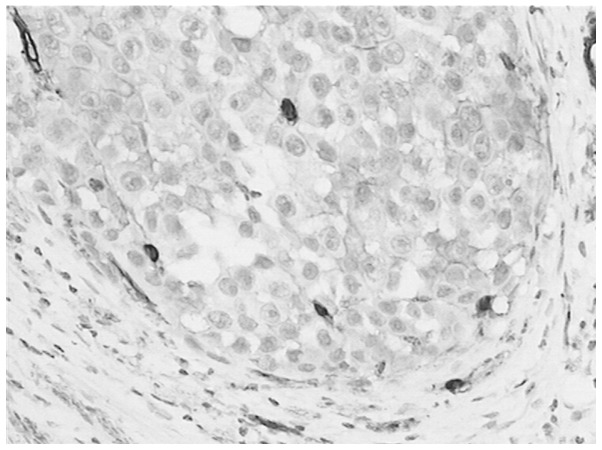
Representative image of weak immunohistochemical staining for CD146 (score of 1) in an esophageal squamous cell carcinoma specimen (magnification, ×200).

**Figure 3:**
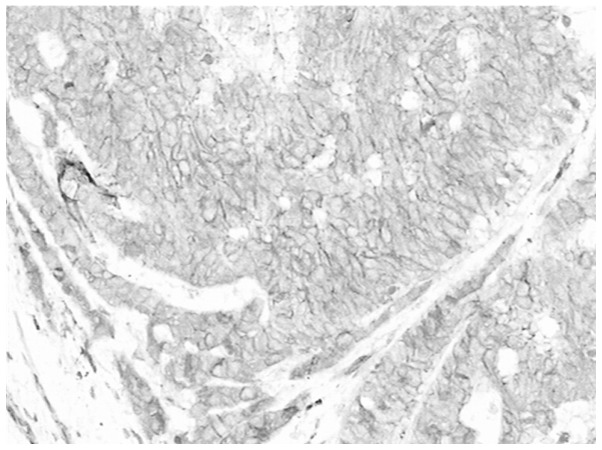
Representative image of weak to moderate immunohistochemical staining for CD146 (score of 2) in an esophageal squamous cell carcinoma specimen (magnification, ×200).

**Figure 4:**
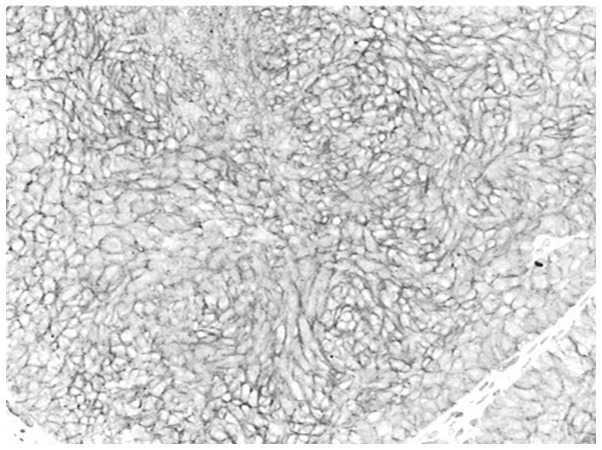
Representative image of moderate to strong immunohistochemical staining for CD146 (score of 3) in an esophageal squamous cell carcinoma specimen (magnification, ×200).

Simultaneously, each section was incubated with PBS instead of the primary antibody as an internal negative control. The immunostained specimens were analyzed by two independent pathologists who were blinded to the patients’ clinicopathological characteristics. Cytoplasm and membrane staining (brown reaction product) was regarded as a positive staining result for CD146. Five fields in each cancer and non-cancer section were evaluated at high power (×400) to determine the proportion of immunostained tumor cells and the staining intensity of the cytoplasm and membrane in the entire sections. The staining strength was graded from 1 to 3: 1, no positive staining or a weak staining (Figs. 1 and 2); 2, weak to moderate staining (Fig 3); 3, moderate to strong staining (Fig. 4). The case with positive cells ≥25% and/or scores ≥2 was considered to be positive (13). At least five fields were observed, the average score in each tumor and non-tumor sections served as the result. All sections were scored twice to confirm the reproducibility of the results, and the highest score from the two observers was reported.

### Follow-up

The patients were followed up every 3 months for the first year and then every 6 months for the next 2 years. The total follow-up period was defined as the time from diagnosis to the date of death or the last follow-up appointment if patients remained alive. All 63 patients were included in survival data analysis. The last follow-up appointment was carried out in May 2013, with a mean observation period of 17.6 months (range, 3–35 months).

### Statistical analysis

All calculations were performed using SPSS software, version 16.0 (SPSS, Inc., Chicago, IL, USA). The associations between CD146 expression and clinicopathological variables were assessed using the χ^2^ and Fisher’s exact tests. The association between CD146 expression and survival time was assessed using the Cox regression model. P<0.05 was considered to indicate a statistically significant difference.

## Results

### Immunohistochemistry

**Figure 5:**
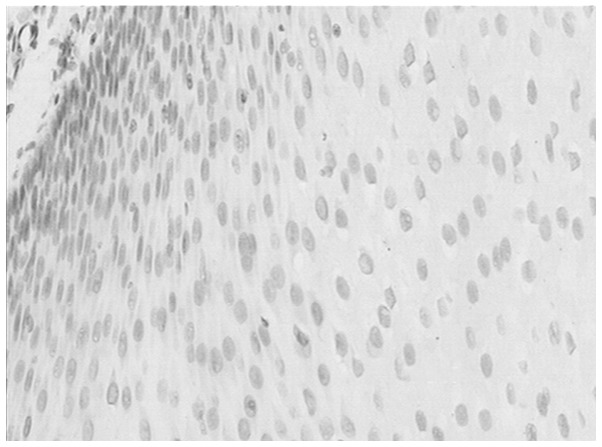
Representative image of negative immunohistochemical staining for CD146 (score of 1) in a normal esophageal mucosa sample (magnification, ×200).

**Figure 6:**
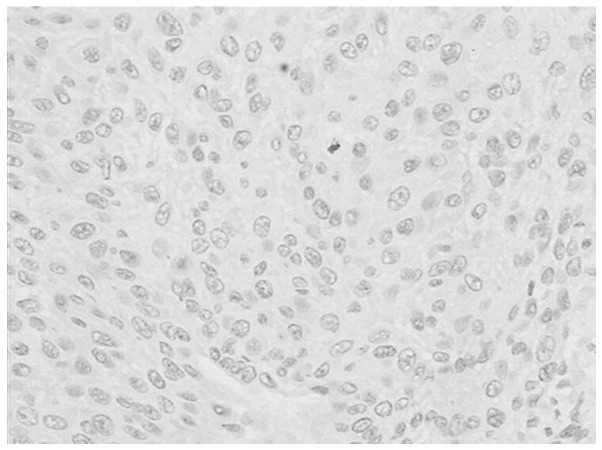
Representative image of phosphate-buffered saline instead of the primary antibody being used as an internal negative control in an esophageal squamous cell carcinoma specimen (magnification, ×200).

Immunohistochemistry revealed that CD146-positive staining was localized in the membrane and cytoplasm of tumor cells in the tumor tissues. CD146 expression was identified in 46.0% (29/63) of the ESCC samples, and no positive (weak to moderate or moderate to strong)expression was found in the 63 normal squamous epithelium samples (Fig. 5) (χ^2^=27.248 P<0.0001). The negative control group was underwent the same steps as previously described, with the exception that the CD146 antibody was replaced with PBS (Fig. 6).

### Associations between CD146 expression and clinicopathological variables

The associations between CD146 expression and clinicopathological variables were investigated. Positive expression of CD146 was found in 46.0% (29/63) of the ESCC samples, which was significantly higher than that in the normal esophageal epithelium samples which demonstrated no immunostaining (χ^2^=27.248; P<0.0001). CD146 expression was associated with lymph node metastasis (χ^2^=5.117; P=0.024) and advanced clinical stage (χ^2^=4.661; P=0.031). No correlation was found with tumor size (χ^2^=2.346; P=0.309), invasion depth (χ^2^=0.962; P=0.327) or tumor differentiation status (χ^2^=1.977; P=0.372) (Table I).

### Association between CD146 expression and survival time

**Figure 7:**
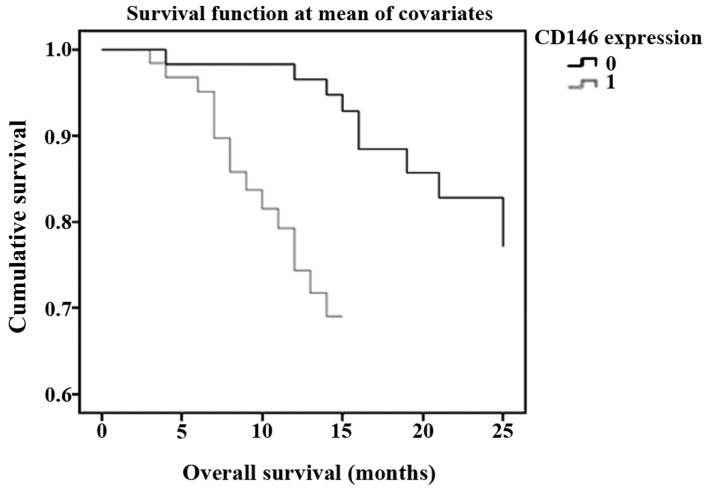
Correlation between CD146 expression and survival time in esophageal squamous cell carcinoma.

The mean survival time of patients with positive CD146 expression was 15 months while that of patients with negative CD146 expression was 25 months. In the multivariate analysis, the association between CD146 expression and survival time was statistically significant (HR, 2.838; 95% CI: 1.102–7.305; P=0.031) (Fig. 7).

## Discussion

CD146 is a cell-cell or cell-matrix adhesion molecule that was first described in melanoma (5). Previous studies have indicated that CD146 expression correlates with the malignant progression and metastatic potential of human melanoma cells (14–18). Expression of CD146 has been observed in certain normal human tissues and numerous malignancies, such as non-small cell lung cancer, gallbladder adenocarcinoma and gastric cancer (13,19,20). The current study demonstrated that CD146 expression was significantly higher in ESCC than in the normal esophageal mucosal tissue. Additionally, it was identified that CD146 expression was associated with lymph node metastasis (P=0.024) and advanced clinical stage (P=0.031) in ESCC. However, no correlations between CD146 expression and tumor size (P=0.309), invasion depth (P=0.327) and differentiation grading (P=0.372) were identified. The results shown in Table I demonstrate that five of the nine low differentiation patients, 19 of the 38 moderate differentiation patients and five of the 16 high differentiation patients were positive for immunohistochemical expression. In addition, the results showed that in tumors with low and intermediate differentiation, the expression of CD146 was higher than that in highly differentiated tumors (55.6, 50.0 and 31.3% respectively), although the differences were not statistically significant. Similarly, 4 of the 12 T2 patients and 25 of the 51 T3 patients were positive for immunohistochemical expression, while five of the 15 patients with tumor sizes of ≤3 cm, 17 of the 37 patients with tumor sizes of 3.1–6 cm and 7 of the 11 patients with tumor sizes of >6 cm were positive for immunohistochemical expression. CD146 expression in T3 tumors (49.0%) was higher than that in T2 tumors (33.3%), and the levels of expression increased with an increase in tumor size (33.3, 45.9, 63.6%), although the results were not statistically significant. The results of the present study demonstrated that CD146 may have a role in malignant progression in esophageal squamous cell carcinoma and may be associated with a more aggressive tumor phenotype. Liu *et al* (20) reported that CD146 expression correlated positively with lymph node involvement in gastric cancer patients. The results of the present study are consistent with this finding in gastric cancer.

CD146 expression has been found to be correlated with aggressiveness and development of metastasis, and is a predictor of worse prognosis in certain cancer types (21). Advanced tumor stage is an important prognostic factor for solid tumors. CD146 is associated with an advanced tumor stage in melanoma, prostate cancer, ovarian cancer and triple-negative breast cancer (7–9,22). In the present study, CD146 was demonstrated to be associated with an advanced tumor stage in ESCC. Metastasis occurs through a series of steps, including local invasion, intravasation, transport, extravasation and colonization (23). Epithelial to mesenchymal transition is a process in which the epithelial cells lose polarity and develop a mesenchymal phenotype and has been implicated in the initiation of metastasis (22). CD146 is a component of the inter-endothelial junction (24), and is now recognized as a marker of mesenchymal cells (25). CD146 may directly or indirectly contribute to tumor aggressiveness by promoting malignant cell motility (10). The presence of lymph node metastasis is an important factor in the clinical evaluation of esophageal cancer patients (26). Lymphangiogenesis is a significant step in the lymphatic metastasis of tumors. Neonatal lymph vessels finally cause metastasis to regional lymph nodes. A previous study has found that lymph vessel density has a close association with progression, metastasis and prognosis of malignant tumors (13). A study by Sundar and Ganesan indicated that tumor-induced lymphangiogenesis was a predictive indicator of metastasis to lymph nodes (27). Tumor-secreted cytokines, such as vascular endothelial growth factors (VEGF)-C and -D, bind to VEGF receptors on lymphatic endothelial cells and induce proliferation and growth of new lymphatic capillaries. This process is similar to the well known mechanism of angiogenesis; the proliferation of new blood vessel capillaries (28). Luo *et al* (14) reported that CD146 directly interacts with actin-linking ezrin-radixin-moesin (ERM) proteins and recruits ERM proteins to cell protrusions, promoting the formation and elongation of microvilli and leading to cytoskeleton remodeling and finally cell migration. However, the exact molecular mechanism whereby CD146 is involved in lymph node metastasis remains unknown. Further studies are required to investigate this issue.

It is well acknowledged that advanced stage and lymph node metastasis are important prognostic factors for ESCC. The current study demonstrated that CD146 expression was associated with advanced clinical stage and lymph node metastasis in ESCC patients, and was therefore an indicator of poor prognosis in these patients. Overexpression of the CD146 gene was one of the important phenotypes and characteristics in ESCC carcinomatous change. This study suggests an important role for CD146 in the development of ESCC. CD146 may present as a potential therapeutic target for the individualized treatment of ESCC.

## Figures and Tables

**Table I:** Correlation between CD146 expression and the clinicopathoclinical parameters in the ESCC group.

Parameters	CD146^+^	CD146^−^	P-value
Age, years			
Range	41–77		
Mean	62.4		
Gender, n			
Male	60		
Female	3		
Tumor size, n			0.309
≤3 cm	5	10	
3.1–6 cm	17	20	
>6 cm	7	4	
Invasion depth^a^, n			0.327
T2	4	8	
T3	25	26	
Differentiation status^b^, n			0.372
Low	5	4	
Moderate	19	19	
High	5	11	
Lymph node metastasis, n			0.024
No	8	19	
Yes	21	15	
Clinical stage^c^, n			0.031
II	10	21	
III	19	13	

a According to the Internation Union against Cancer 2002 (12);

b according to World Health organization criteria 2000 (11); and

c according to the tumor-node-metastasis classification system of the International Union against Cancer (12).

ESCC, esophageal squamous cell carcinoma.
